# Effect of Perioperative Interleukin-6 and Tumor Necrosis Factor-*α* on Long-Term Outcomes in Locally Advanced Gastric Cancer: Results from the CLASS-01 Trial

**DOI:** 10.1155/2022/7863480

**Published:** 2022-07-08

**Authors:** Zhenzhan Zhang, Binshu Weng, Yaopeng Qiu, Hao Feng, Renyi Zhang, Jiachen Zhang, Yanfeng Hu, Jiang Yu, Guoxin Li, Hao Liu

**Affiliations:** ^1^Department of General Surgery, Nanfang Hospital, Southern Medical University, Guangzhou, China; ^2^Clinical Research Center, Nanfang Hospital, Southern Medical University, Guangzhou, China

## Abstract

**Background:**

Little is known about the relation between perioperative inflammatory changes and long-term survival in cancer patients. The aim of the study was to assess the association of perioperative serum interleukin-6 (IL6) and tumor necrosis factor-*α* (TNF*α*) levels with the 5-year overall survival in locally advanced gastric cancer.

**Methods:**

The 135 eligible patients in one center of Nanfang Hospital were retrieved from CLASS-01 trial (NCT01609309), an open-label, multicenter, randomized clinical noninferiority trial conducted at 14 centers in China. Serum IL6 and TNF*α* levels were tested before surgery, and on postoperative day (POD) 1, POD3, and POD5, respectively, referring to IL6_0, IL6_1, IL6_3, and IL6_5 and TNF*α*_0, TNF*α*_1, TNF*α*_3, and TNF*α*_5. Kaplan-Meier methods and COX models were used for survival analysis.

**Results:**

High levels of IL6_0 (≥3.67 pg/mL) and TNF*α*_0 (≥14.8 pg/mL) presented worse disease-free survival (DFS) (*P* = 0.0057 for IL6_0 and *P* = 0.0014 for TNF*α*_0) and overall survival (OS) (*P* = 0.0021 for IL6_0 and *P* = 0.0019 for TNF*α*_0). Both high IL6_0 and high IL6_5 levels indicated worse prognosis than other combinations (*P* = 0.0045 for DFS and *P* = 0.0022 for OS). In multivariate analysis, both high IL6_0 and high IL6_5 levels were significantly associated with poor DFS (HR = 4.29, 95% CI: 1.42-12.95, *P* = 0.01) and OS (HR = 4.11, 95% CI: 1.35-12.49, *P* = 0.013) after adjustment of tumor stage and TNF*α*_0. Also, high IL6_5 level was identified as the independent-related factor for postoperative infectious complications (OR = 2.69, 95% CI: 1.03-7.01, *P* = 0.043).

**Conclusions:**

Perioperative high serum IL6 and TNF*α* levels are negatively associated with 5-year survival outcomes in patients with locally advanced gastric cancer, indicating the potential survival benefits from perioperative anti-inflammatory treatment.

## 1. Introduction

The perioperative period is critical in determining the long-term survival outcomes in cancers [1–5]. Specifically, perioperative inflammation state plays a pivotal role as deleterious mediators impacting on surgical short-term and long-term prognosis [5]. Levels of preoperative inflammatory biomarkers represent the systemic inflammatory status of patients before surgery. Inflammation predisposes to the development of cancer and promotes all stages of tumorigenesis. Indeed, almost 15%–20% of cancer cases are preceded by infection, chronic inflammation, or autoimmunity [6, 7], and the most prominent examples include inflammatory bowel disease, chronic hepatitis, and Helicobacter-induced gastritis, increasing the risk of colorectal cancer, liver cancer, and stomach cancer, respectively, [8]. In addition, extensive preclinical researches have shown that the stress-inflammatory response triggered by surgery and immunosuppression promotes the new metastases and increases the cancer-related mortality [1, 4, 9]. Accordingly, perioperative inflammatory response should be taken seriously in therapeutic management for gastric cancer.

There is a long history of anti-inflammatory treatment for patients with different cancers, albeit in the absence of direct clinical evidence of effectiveness for survival benefit [10]. The clinical use of anti-inflammatory therapy in cancer during the perioperative timeframe has been less frequently studied with contradictory results [10–12]. Most previous studies only assessed inflammatory markers preoperatively or several months later [13–15] rather than focused on monitoring the whole changes of perioperative inflammatory factor levels, contributing few evidences for perioperative anti-inflammatory managements. More researches exploring the association between alteration of perioperative inflammatory biomarkers levels and cancer prognosis are needed to provide evidences for perioperative anti-inflammatory treatment.

Regarding that traditional radiotherapy and chemotherapy are inappropriate to be exerted during perioperative timeframe given their suppressive effects on the immune system and tissue healing [5], anti-inflammatory therapy may help in developing novel perioperative therapeutic strategies upon adjuvant radiotherapy and chemotherapy in cancer. Inflammatory responses were secondary end-points for CLASS-01 trial, a noninferiority, open-label, randomized clinical trial at 14 centers in China, based on which we have confirmed the short-term and long-term safety of radical laparoscopic distal gastrectomy with D2 lymphadenectomy for the treatment of locally advanced gastric cancer (LAGC) [16–18]. Interleukin-6 (IL6) and tumor necrosis factor-*α* (TNF*α*), as two of the typical inflammatory factors to regulate key process of inflammation and immune response, could reflect the whole inflammatory status in the human body. Additionally, the serum levels of IL6 and TNF*α* were easy to be detected in human peripheral blood. In present study, we report IL6 and TNF*α* to investigate the association of perioperative serum levels of them with long-term outcomes of LAGC with radical gastrectomy, aiming to provide a rationale for developing perioperative anti-inflammatory therapeutic strategies.

## 2. Materials and Methods

### 2.1. Study Design and Population

The CLASS-01 trial (ClinicalTrials.gov Identifier: NCT01609309) was an open-label, multicenter, randomized clinical noninferiority trial conducted at 14 centers in China [16–18]. The approved study protocol and statistical analysis plan are available in the CLASS-01 trial. All patients provided informed consent for obtaining specimens, and the study was approved by the Clinical Research Ethics Committees of the Nanfang Hospital, Southern Medical University. Patients were enrolled from September 12, 2012, through December 3, 2014. The trial enrolled patients if they were aged 18 to 75 years; had histologically confirmed gastric adenocarcinoma, detected at a locally advanced stage, according to the Japanese Classification [17] (and to T2-4aN0-3M0, corresponding to stages IB-IIIC excluding T1 or T4b tumors); had tumors located in the lower or middle third of the stomach by preoperative evaluation; and were expected to undergo distal gastrectomy with D2 lymphadenectomy for curative intent. Clinical and pathological TNM stages were according to the Cancer Staging Manual, 7th edition [19]. Patients with previous neoadjuvant chemotherapy or radiotherapy were excluded. The details of the other exclusion criteria can refer to the study protocol of CLASS-01 trial. Inflammatory biomarkers were primary research targets in this study: serum IL6 and TNF*α* were tested before surgery, and on postoperative day (POD) 1, POD3, and POD5, respectively, referring to IL6_0, IL6_1, IL6_3, and IL6_5 and TNF*α*_0, TNF*α*_1, TNF*α*_3, and TNF*α*_5. The detection of human serum IL6 and TNF*α* levels was used by the Human IL-6 Quantikine ELISA Kit (#D6050, R&D Systems, USA) and the Human TNF-alpha Quantikine ELISA Kit (#DTA00D, R&D Systems, USA) according to the manufacturer's instructions. The final follow-up was on March 31, 2020. The 135 patients in one center of Nanfang Hospital testing inflammatory biomarkers at fixed time points during perioperative period were analyzed.

### 2.2. Outcomes

Disease-free survival (DFS) and overall survival (OS) were primary endpoints in this study. Patients were followed up for a minimum of 60 months. OS was calculated from the day of randomization until the day of death (event) or the day of the last follow-up examination (censored). DFS was calculated from the day of randomization until the day of recurrence (event) or death (event) or the day of the last follow-up examination (censored). Data were censored for patients with no evidence of disease at the last follow-up examination.

### 2.3. Statistical Analysis

We used receiver operator characteristic (ROC) curve to analyze the relationship of serum levels of IL6_0, IL6_1, IL6_3, IL6_5, TNF*α*_0, TNF*α*_1, TNF*α*_3, and TNF*α*_5 with 5-year OS status and calculated their optimal cut-off values for predicting the outcomes of survival. High and low levels of inflammation markers of serum IL6 and TNF*α* were divided according to the optimal cut-off values. Cox proportional hazards regression analysis was used to determine and adjust the effect of levels of serum IL6 and TNF*α* on DFS and OS. We used the Kaplan-Meier method to estimate the differences between the high levels and low levels of serum IL6 and TNF*α* in DFS and OS. Furthermore, logistic regression model was used to estimate the association of important clinical characteristics with complications. All analyses were performed using SPSS, version 25 (IBM Corp), and R, version 4.0.2 (R Group for Statistical Computing). *P* < 0.05 was considered significant.

### 2.4. Role of the Funding Source

The funder of the study had no role in study design, data collection, data analysis, data interpretation, or writing of the report. The corresponding authors had full access to all the data in the study and had final responsibility for the decision to submit for publication.

## 3. Results

### 3.1. Study Population

**Figure 1:**
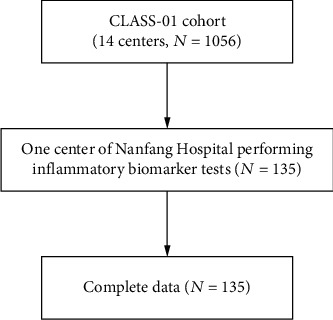
The flowchart of population.

A total of 135 patients were eligible to be enrolled in this trial from September 12, 2012, through December 3, 2014 (Figure 1). The characteristics of the patients are shown in Table 1. Mean age of the population was 52.5 years (SD, 11.4 years), and 84 (62.2%) patients were men. Of 135 patients, 67 underwent laparoscopic gastrectomy while 68 underwent open gastrectomy. While clinical TNM stage evaluated before operation was consisted of 6 (4%) in stage I, 59 (44%) in stage II, and 70 (52%) in stage III, the pathological TNM stage was consisted of 44 (33%) in stage I, 46 (34%) in stage II, and 45 (33%) in stage III after confirmed by surgical pathology. There were 29 patients presenting with complications, among which 24 patients presented with postoperative infectious complications including 8 patients with pulmonary infections, 8 with lymphorrhagia, 7 with effusion or pyocelia, and 3 with urinary tract infection. Eight patents had other complications. Among the population, 65 patients accepted adjuvant chemotherapy. Table 1 shows the preoperative basic inflammation conditions and target inflammation markers at different time points.

### 3.2. Inflammation Markers Predicting Five-Year OS Outcomes

We used ROC curve to analyze the association of IL6 and TNF*α* with 5-year OS status (Supplementary Table 1). Area under the curve (AUC, 95% confidence interval (CI)), *P* value, optimal cut-off value, sensitivity, and specificity were all calculated for each factor, among which IL6_0 (AUC = 0.655, 95% CI: 0.549-0.761) and TNF*α*_0 (AUC = 0.635, 95% CI: 0.518-0.751) showed significant value for predicting the outcome of 5-year OS. The optimal cut-off value of IL6_0 was 3.67 pg/mL, with sensitivity of 86.2% and specificity of 46.4%. When applying this optimal cut-off value to predict 5-year DFS, the sensitivity and specificity remained 79.4% and 45.7%, respectively. The optimal cut-off value of TNF*α*_0 was 14.8 pg/mL, with sensitivity of 64.3% and specificity of 68.8%, and when it went to 5-year DFS, the sensitivity and specificity were 65.5% and 69.5%, respectively. The optimal cut-off values were used to define high levels and low levels of IL6_0 (high IL6_0: ≥3.67 pg/mL; low IL6_0: <3.67 pg/mL) and TNF*α*_0 (high TNF*α*_0: ≥14.8 pg/mL; low TNF*α*_0: <14.8 pg/mL). The classification of other inflammatory factors was also liked IL6_0 and TNF*α*_0.

### 3.3. Survival Analysis

**Figure 2:**
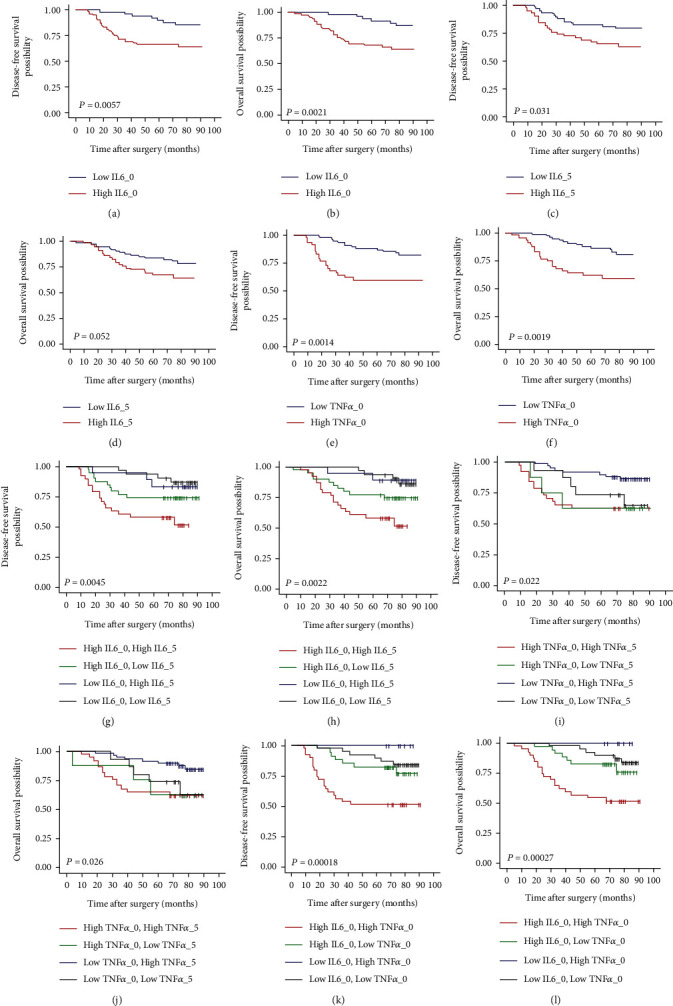
(a) The comparison of DFS between high and low IL6_0 levels. (b) The comparison of OS between high and low IL6_0 levels. (c) The comparison of DFS between high and low IL6_5 levels. (d) The comparison of OS between high and low IL6_5 levels. (e) The comparison of DFS between high and low TNF*α*_0 levels. (f) The comparison of OS between high and low TNF*α*_0 levels. (g) The combination analysis of IL6_0 and IL6_5 in DFS. (h) The combination analysis of IL6_0 and IL6_5 in OS. (i) The combination analysis of TNF*α*_0 and TNF*α*_5 in DFS. (j) The combination analysis of TNF*α*_0 and TNF*α*_5 in OS. (k) The combination analysis of IL6_0 and TNF*α*_0 in DFS. (l) The combination analysis of IL6_0 and TNF*α*_0 in OS.

The median follow-up period was 76 months (IQR, 66-81 months), with 27 recurrences and 36 deaths. The rates of 5-year cumulative DFS and OS were 73% and 75%, respectively. In Kaplan-Meier curve analysis, high IL6_0 presented poorer survival in both DFS (*P* = 0.0057, Figure 2(a)) and OS (*P* = 0.0021, Figure 2(b)). High level of IL6_5 indicated poorer prognosis in DFS (*P* = 0.031, Figure 2(c)), but the impact on OS were not able to reach statistical significance with *P* = 0.052 (Figure 2(d)). High TNF*α*_0 also showed negative survival in both DFS (*P* = 0.0012, Figure 2(e)) and OS (*P* = 0.0019, Figure 2(f)). The factors of IL6_1, IL6_3, TNF*α*_1, TNF*α*_3, and TNF*α*_5 did not show robust connection with DFS and OS (Supplementary Figure 1). In order to have a more comprehensive understanding of the association of perioperative inflammatory states with long-term outcomes, the nonlinear association between the serum IL6_0 and TNF*α*_0 levels with survival in patients with gastric cancer was explored (Supplementary Figure 2). We combined IL6_0 and IL6_5 to explore the correlation to prognosis (group A: high IL6_0 and high IL6_5; group B: high IL6_0 and low IL6_5; group C: low IL6_0 and high IL6_5; group D: low IL6_0 and low IL6_5), as a result of which the group A showed worst survival of all (*P* = 0.0045 in DFS, Figure 2(g); *P* = 0.0022 in OS, Figure 2(h)) and significantly worse than the group B (*P* = 0.044 in DFS and *P* = 0.058 in OS). Additionally, we also combined TNF*α*_0 and TNF*α*_5 to analyze survival. The group of high TNF*α*_0 and high TNF*α*_5 also seemed to have a worse prognosis (*P* = 0.022 in DFS, Figure 2(i); *P* = 0.026 in OS, Figure 2(j)). Furthermore, we combined factors of IL6_0 with TNF*α*_0 to demonstrate interactive relationships between their levels and survival (group 1: high IL6_0 and high TNF*α*_0; group 2: high IL6_0 and low TNF*α*_0; group 3: low IL6_0 and high TNF*α*_0; group 4: low IL6_0 and low TNF*α*_0), showing that group 1 indicated poorer prognosis than other groups (*P* = 0.00018 in DFS, Figure 2(k); *P* = 0.00027 in OS, Figure 2(l)). Univariate analysis demonstrated that pathological stage of III (hazard ratio (HR) = 6.15, 95% CI: 3.02-12.54, *P* < 0.001), high IL6_0 (HR = 3.54, 95% CI: 1.46-8.60, *P* = 0.005), and high TNF*α*_0 (HR = 3.21, 95% CI: 1.56-6.63, *P* = 0.002) were significantly correlated with DFS. Variables with *P* < 0.05 in univariate analysis were included in multivariate analysis, showing that pathological tumor stage of III (HR = 5.40, 95% CI: 2.46-11.86, *P* < 0.001) and high IL6_0 (HR = 2.98, 95% CI: 1.11-7.98, *P* = 0.03) were independent prognostic factors for DFS (Table 2). In univariate analysis of OS, pathological tumor stage of III (HR = 6.17, 95% CI: 3.03-12.58, *P* < 0.001), high IL6_0 (HR = 3.67, 95% CI: 1.51-8.92, *P* = 0.004), and high TNF*α*_0 (HR = 2.91, 95% CI: 1.44-5.89, *P* = 0.003) also showed significantly correlation with outcome, and as a result of multivariate analysis, pathological tumor stage of III (HR = 5.00, 95% CI: 2.34-10.67, *P* < 0.001) and high IL6_0 (HR = 2.67, 95% CI: 1.06-6.72, *P* = 0.037) were independent prognostic factors for OS (Table 2). In multivariate analysis of combination of IL6_0 and IL6_5, group A was the independent risk factor for both DFS (HR = 4.29, 95% CI: 1.42-12.95, *P* = 0.010) and OS (HR = 4.11, 95% CI: 1.35-12.49, *P* = 0.013) after adjusted by tumor stage and TNF*α*_0 (Table 3). Furthermore, based on the stratified analysis at tumor stage (stages I/II/III), we observed that LAGC patients with high levels of IL6_0 and TNF*α*_0 at advanced tumor stage have poor prognosis by the description of Kaplan-Meier curves (Supplementary Figure 3).

### 3.4. Analysis for Complications

Finally, we next evaluated the associations between levels of inflammatory markers and complications. Of total complications, inflammatory markers did not show significantly relevant. Of postoperative infectious complications, ECOG score of 1 (OR = 2.28, 95% CI: 0.91-5.74, *P* = 0.080), BMI ≥ 25 kg/m^2^ (OR = 2.64, 95% CI: 0.94-7.42, *P* = 0.066), and high IL6_5 (OR = 2.48, 95% CI: 1.00-6.18, *P* = 0.051) with *P* < 0.10 in univariate analysis were included in multivariate analysis, in which ECOG score of 1 (OR = 2.75, 95% CI: 1.03-7.32, *P* = 0.043), BMI ≥ 25 kg/m^2^ (OR = 3.42, 95% CI: 1.12-10.46, *P* = 0.031), and high IL6_5 (OR = 2.69, 95% CI: 1.03-7.01, *P* = 0.043) remained to be independent-related factors for postoperative infectious complications (Table 4). We further analyzed the internal relationship between IL6_5 level and specific complications (Supplementary Table 2). Occurrences of the pulmonary infections and Clavien–Dindo classification of II in group of high IL6_5 were more than group of low IL6_5 (10.4% vs. 1.4%, *P* = 0.044, and 23.6% vs. 13.7%, *P* = 0.048, respectively).

## 4. Discussion

In present study, serum IL6 and TNF*α* levels increased after operation and then decreased gradually. High levels of perioperative serum IL6 and TNF*α* levels were associated with worse survival in LAGC patients. Especially, both high levels of preoperative IL6 level and IL6 level on postoperative day 5 were an independent risk factor for survival. High IL6 level on postoperative day 5 was an independent-related factor for postoperative infectious complications. The findings would provide potential for anti-inflammatory treatment during perioperative period. Previous studies have reported the association of inflammatory factors with survival outcomes in specific cancers [14, 20, 21]. Preoperative elevated IL6 levels have been reported to be associated with poorer prognosis in gastric cancer [20, 21], which was in line with parts of results in our study, but they only focused on IL6 levels before surgery without testing IL6 at several time points like we did. Wesselink et al. estimated multiple inflammatory markers of IL6, IL-8, IL-10, TNF*α*, CRP, and a combined inflammatory *z*-score before and 6 months after surgery in colorectal cancer and found that higher CRP, IL-8, and combined inflammatory *z*-score levels before surgery and 6 months later were associated with a higher risk of recurrence and mortality [14], which was a relatively comprehensive study on the association of inflammatory states with survival outcomes, but inflammatory markers were not tested postoperatively during perioperative timeframe, providing little for perioperative anti-inflammatory management. In another study testing the changes of inflammatory factor levels after major abdominal surgery, the authors found IL6 levels more than 432 pg/mL on the postoperative day 1 which were associated with an increased risk of postoperative complications [22], but regrettably, the research did not involve the pivotal point of survival analysis.

Importantly, cancer patients may benefit from perioperative anti-inflammatory therapy in terms of improved long-term survival. An Israeli team has conducted two clinical randomized trials to assess the efficacy of perioperative anti-inflammatory therapy in breast and colorectal cancers, respectively, both finding that epithelial-to-mesenchymal transition was reduce and immune microenvironment was improved in transcriptome profiling of the primary tumor, indicating lower three-year recurrence rates in colorectal cancer [11, 12]. In present study, the preoperative basic inflammatory states were generally within normal ranges. When patients with increased preoperative IL6 levels but decreased postoperative IL6 levels on fifth day after surgery, the outcomes were much better than those with both high levels, which indicated that perioperative anti-inflammatory therapy for patients with preoperative elevated IL6 levels may decrease IL6 levels perioperatively and may contribute to improved long-term survival. Accordingly, we would like to conduct a prospective clinical trial to validate the hypothesis that whether patients with preoperative elevated inflammatory levels treated with perioperative anti-inflammatory therapy would improve their inflammatory states postoperatively and ultimately obtain long-term survival in LAGC.

Interestingly, combined preoperative IL6 and TNF*α* may have synergetic effect on serving as survival predictor. Preclinical vitro studies provided evidences that TNF*α* can induce Activin-A to enhance the mRNA expression of IL6 [23] and also induce IL6 synthesis through the JAK/STAT3 pathway in addition to p38 MAP kinase and SAPK/JNK [24]. Furthermore, a retrospective clinical study involving 144 hospitalized patients with cancers showed that simultaneously elevated IL6 and TNF*α* levels had a nearly 6-fold increase in mortality [25]. Consistently, in our study, we also found that both preoperative high IL6 and high TNF*α* levels had an about 4.4-fold increase in mortality compared with both preoperative low IL6 and low TNF*α* levels in LAGC. Considering that IL6 and TNF*α* have a close interaction and play essential roles in inflammatory response and cancer long-term outcomes, the combination of them seems to be a potentially more accurate, predictive, and sensitive indicator for predicting cancer prognosis.

Compared with factors of IL6, TNF*α* seems to be less determinate in predicting the outcomes of cancer survival. TNF*α* can have both pro- and antitumorigenic effects based on interaction with its receptors TNFR1 and TNFR2 in cancer [26]. In a meta-analysis including 20 articles and involving 11094 patients, TNF*α* rs361525 polymorphism was proved to link to the risk of gastric cancer [27]. But in the other clinical study involving 71 operative gastric cancer cases, preoperative high TNF*α* indicated better outcomes [28]. Although both preoperative IL6 and TNF*α* showed important roles in predicting the prognosis of gastric cancer patients in the present study, preoperative TNF*α* was not the independent prognostic factor for DFS and OS after adjustment for preoperative IL6 and tumor stage, while preoperative IL6 was the independent prognostic factor after adjusted with preoperative TNF*α* and tumor stage.

Postoperative IL6 levels may link to postoperative infectious complications. In a prospective single-center study, IL6, TNF*α*, CRP, and systemic inflammatory response syndrome were tested several time points after abdominal surgery to measure associations with postoperative complications, not exactly the same to our results, finding that high IL6 levels on postoperative day 1 increased the risk for postoperative complication [22]. Nevertheless, Moris et al. [29] held a different perspective that IL6 was important but not sufficient to predict morbidity after a major abdominal surgery, considering that postoperative immunosuppression, sympathetic/adrenomedullary system, and hypothalamic-pituitary-adrenal axis were also indispensable for indicating postoperative complications. In our study, we did not find an association of perioperative IL6 and TNF*α* levels with overall postoperative complications, but in specific subgroup of infectious complications, we found that high levels of IL6 on postoperative day 5 might indicate occurrences of postoperative infectious complications. Additionally, our previous study suggested that the inflammatory marker of granulocyte-to-lymphocyte ratio on postoperative day 5 was an independent factor for postoperative infectious complication in in LAGC [30]. Accordingly, the inflammatory factors on postoperative day 5 may have a robust relationship with postoperative infectious complications, suggesting intensive monitoring may help early detection of postoperative complications.

Our study has strengths that this trial prospectively collected inflammatory factors responding to surgical stress and tested inflammatory biomarkers at fixed time points during perioperative period, providing comprehensive information for focusing the research on the association of perioperative inflammatory states with survival in LAGC and providing potential value in suggesting anti-inflammatory treatment during perioperative period while the treatment of chemotherapy and radiotherapy is absent in this period. And we have a long-term survival with the median follow-up period of 76 months, which was convincing to evaluate outcomes in LAGC. Moreover, besides long-term outcomes, we also recorded the details of short-term outcomes of LAGC patients undergoing gastrectomy for better understanding the relationship between perioperative inflammation and postoperative complications. Finally, we found that combined indicator of preoperative IL6 with preoperative TNF*α* may have greater value than either of them in predicting long-term outcomes in LAGC with radical gastrectomy. Our study also has some limitations. Firstly, only Nanfang Hospital tested IL6 and TNF*α* rather than all the centers in CLASS-01, but considering that this is a well-designed, prospective trial with long-term survival and few missing outcomes during follow-up period, the results of this study are convincing. Secondly, the monitoring of inflammatory factors only lasted until the fifth postoperative day rather than longer term after discharge, but we have measured the inflammatory factors several times at fixed time points, comprehensively monitoring the dynamic changes of inflammatory biomarkers during perioperative period. Thirdly, although IL6 and TNF*α* are typical inflammatory biomarkers and we have deeply explored the value of them, it would be better if we would have tested more inflammatory factors, such as IL-8, IL-10, TNF-*β*, with which we could estimate inflammatory conditions more comprehensively.

## Figures and Tables

**Table 1:** Baseline characteristics of patients with gastric cancer.

Characteristics	*N* = 135	%
Age (years, mean ± SD)	52.5 ± 11.4	
Gender		
Female	51	38
Male	84	62
ECOG score		
0	97	72
1	36	27
Missing	2	1
BMI (kg/m^2^, WHO)		
< 25	133	84
≥ 25	22	16
Clinical TNM stage		
I	6	4
II	59	44
III	70	52
Types of surgery		
Laparoscopic gastrectomy	67	50
Open gastrectomy	68	50
Pathological TNM stage		
I	44	33
II	46	34
III	45	33
Complication		
Yes	29	22
No	106	79
Chemotherapy		
Yes	65	48
No	70	52
White blood cell (10^9^/L, median, IQR)	5.89 (4.90-7.02)	
Neutrophil (10^9^/L, median, IQR)	3.34 (2.64-4.17)	
Lymphocyte (10^9^/L, median, IQR)	1.90 (1.61-2.22)	
C-reactive protein (mg/L, median, IQR)	0.9 (0.3-2.1)	
IL6_0 (pg/mL, median, IQR)^a^	5.95 (2.50-14.8)	
IL6_1 (pg/mL, median, IQR)^a^	70.90 (40.28-130.28)	
IL6_3 (pg/mL, median, IQR)^a^	39.65 (17.83-75.10)	
IL6_5 (pg/mL, median, IQR)^a^	18.50 (10.50-43.10)	
TNF*α*_0 (pg/mL, median, IQR)^a^	11.40 (8.35-19.45)	
TNF*α*_1 (pg/mL, median, IQR)^a^	12.10 (9.20-21.10)	
TNF*α*_3 (pg/mL, median, IQR)^a^	16.50 (13.00-24.00)	
TNF*α*_5 (pg/mL, median, IQR)^a^	15.00 (11.00-13.00)	

The IL6_0 was tested preoperatively, and the factors of IL6_1, IL6_3, and IL6_5 were tested on first, third, and fifth days after operation, separately. The TNF*α*_0 was tested preoperatively, and the factors of TNF*α*_1, TNF*α*_3, and TNF*α*_5 were tested on first, third, and fifth days after operation, separately.

**Table 2:** Multivariate analysis for predictors of DFS and OS.

Characteristics	DFS				OS			
	Univariate analysis^a.^		Multivariate analysis		Univariate analysis^a.^		Multivariate analysis	
	HR, 95% CI	*P* value	HR, 95% CI	*P* value	HR, 95% CI	*P* value	HR, 95% CI	*P* value
Age (years)								
<65	Ref.				Ref.			
≥65	0.79 (0.24-2.56)	0.69			0.78 (0.24-2.56)	0.687		
Gender								
Female	Ref.				Ref.			
Male	0.89 (0.46-1.74)	0.738			0.80 (0.41-1.55)	0.512		
ECOG score								
0	Ref.				Ref.			
1	0.81 (0.37-1.79)	0.608			0.82 (0.37-1.80)	0.62		
BMI (kg/m^2^)								
< 25	Ref.				Ref.			
≥ 25	1.21 (0.53-2.76)	0.651			1.19 (0.52-2.71)	0.682		
Types of surgery								
Laparoscopic gastrectomy	Ref.				Ref.			
Open gastrectomy	0.67 (0.35-1.31)	0.244			0.67 (0.35-1.31)	0.243		
Pathological TNM stage								
I-II	Ref.				Ref.			
III	6.15 (3.02-12.54)	<0.001	5.40 (2.460-11.86)	<0.001	6.17 (3.3-12.58)	<0.001	5.00 (2.34-10.67)	<0.001
Complication								
No	Ref.				Ref.			
Yes	0.87 (0.38-1.99)	0.741			0.89 (0.39-2.03)	0.781		
Chemotherapy								
No	Ref.				Ref.			
Yes	1.42 (0.73-2.73)	0.301			1.35 (0.70-2.61)	0.367		
IL6_0								
Low	Ref.				Ref.			
High	3.54 (1.46-8.60)	0.005	2.98 (1.11-7.98)	0.030	3.67 (1.51-8.92)	0.004	2.67 (1.06-6.72)	0.037
TNF*α*_0								
Low	Ref.				Ref.			
High	3.21 (1.56-6.63)	0.002	1.93 (0.91-4.07)	0.086	2.91 (1.44-5.89)	0.003	1.73 (0.83-3.62)	0.144

^a^Variables with *P* < 0.05 in univariate analysis were included in multivariate analysis.

**Table 3:** Multivariate analysis of combined factors of IL6_0 and IL6_5 with DFS and OS.

Characteristics	DFS				OS			
	Univariate analysis^b^		Multivariate analysis		Univariate analysis^b^		Multivariate analysis	
	HR, 95% CI	*P* value	HR, 95% CI	*P* value	HR, 95% CI	*P* value	HR, 95% CI	*P* value
Pathological TNM stage								
I-II	Ref.				Ref.			
III	6.15 (3.02-12.54)	<0.001	5.40 (2.46-11.86)	<0.001	6.17 (3.03-12.58)	<0.001	5.00 (2.34-10.67)	<0.001
Combination of IL6_0 and IL6_5								
Group A^a^	4.88 (1.64-14.54)	0.004	4.29 (1.42-12.95)	0.010	5.00 (1.67-14.95)	0.004	4.11 (1.35-12.49)	0.013
Group B^a^	2.29 (0.72-7.30)	0.162	1.41 (0.42-4.73)	0.582	2.40 (0.75-7.66)	0.140	1.62 (0.48-5.46)	0.439
Group C^a^	1.35 (0.30-6.04)	0.694	1.15 (0.21-6.29)	0.871	0.91 (0.17-4.98)	0.916	1.15 (0.21-6.30)	0.869
Group D^a^	Ref.				Ref.			
TNF*α*_0								
Low	Ref.				Ref.			
High	3.21 (1.56-6.63)	0.002	1.93 (0.91-4.07)	0.086	2.91 (1.44-5.89)	0.003	1.73 (0.83-3.62)	0.144

^a^Group A: high IL6_0 and high IL6_5 levels; group B: high IL6_0 and low IL6_5 levels; group C: low IL6_0 and high IL6_5 levels; group D: low IL6_0 and low IL6_5 levels. ^b^Variables with *P* < 0.05 in univariate analysis were included in multivariate analysis.

**Table 4:** Multivariate analysis for complications.

Characteristics	Total complications				Infectious complications			
	Univariate analysis^a^		Multivariate analysis		Univariate analysis^a^		Multivariate analysis	
	OR, 95% CI	*P* value	OR, 95% CI	*P* value	OR, 95% CI	*P* value	OR, 95% CI	*P* value
Age (years, ≥ 65)	1.54 (0.40-5.03)	0.498			1.30 (0.33-5.06)	0.707		
Gender (male)	2.23 (0.91-6.05)	0.092	2.11 (0.81-5.54)	0.127	1.60 (0.61-4.16)	0.34		
ECOG score [1]	1.93 (0.79-4.60)	0.14			2.28 (0.91-5.74)	0.08	2.75 (1.03-7.32)	0.043
BMI (kg/m^2^, ≥ 25)	2.50 (0.90-6.66)	0.069	2.87 (1.01-8.16)	0.048	2.64 (0.94-7.42)	0.066	3.42 (1.12-10.46)	0.031
Types of surgery (open gastrectomy)	0.63 (0.27-1.44)	0.277			0.80 (0.33-1.94)	0.624		
Pathological TNM stage (III)	1.56 (0.66-3.62)	0.302			1.55 (0.63-3.84)	0.342		
Chemotherapy (yes)	1.20 (0.53-2.75)	0.664			1.34 (0.55-3.25)	0.561		
IL6_0 (high)	1.56 (0.64-4.12)	0.343			2.31 (0.79-6.77)	0.128		
IL6_1 (high)	2.07 (0.77-6.60)	0.178			2.08 (0.66-6.61)	0.213		
IL6_3 (high)	1.75 (0.65-5.61)	0.3			1.80 (0.56-5.71)	0.322		
IL6_5 (high)	2.11 (0.92-4.97)	0.081	2.07 (0.87-4.96)	0.101	2.48 (1.00-6.18)	0.051	2.69 (1.03-7.01)	0.043
TNF*α*_0 (high)	0.54 (0.20-1.38)	0.216			0.58 (0.21-1.61)	0.292		
TNF*α*_1 (high)	0.87 (0.33-2.45)	0.777			0.85 (0.30-2.43)	0.777		
TNF*α*_3 (high)	0.65 (0.22-1.70)	0.402			0.67 (0.23-2.03)	0.496		
TNF*α*_5 (high)	0.46 (0.18-1.22)	0.11			0.70 (0.25-1.99)	0.502		

^a^Variables with *P* < 0.1 in univariate analysis were included in multivariate analysis.

## Data Availability

The original data can be available if readers turn to the corresponding authors with reasonable grounds.

## Supplementary Materials

Supplementary 1 Table 1: the analysis of ROC curve of the relationship between inflammatory markers and 5-year OS. Table 2: the association of IL6_5 levels with short-term outcomes.

Supplementary 2 Figure 1: the association of IL6_1, IL6_3, TNF*α*_1, TNF*α*_3, and TNF*α*_5 levels with DFS and OS in LAGC. Figure 2: the nonlinear association between the serum IL6_0 and TNF*α*_0 levels with survival in LAGC patients. Figure 3: the stratified analysis of IL6_0, TNF*α*_0, and survival prognosis based on the TNM stage (stages I/II/III).
