# Supplementary material for: Effect of Perioperative Interleukin-6 and Tumor Necrosis Factor-α on Long-Term Outcomes in Locally Advanced Gastric Cancer: Results from the CLASS-01 Trial
Source: J Immunol Res. 2022 Jul 8;2022:7863480. doi: 10.1155/2022/7863480 (PMC9289757; doi:10.1155/2022/7863480)
Supplement: Supplementary 1 — Table 1: the analysis of ROC curve of the relationship between inflammatory markers and 5-year OS. Table 2: the association of IL6_5 levels with short-term outcomes. [file 7863480.f1.zip › 7863480.f1/Supplementary Table 1.pdf]

Supplementary Table 1 The analysis of ROC curve of the relationship between inflammatory markers and 5-year OS

Inflammatory markers	AUC，95% CI	P value	Optimal cut-off value, pg/mL	Sensitivity	Specificity
IL6_0	0.655 (0.549 - 0.761)	0.012	3.67	86.20%	46.40%
IL6_1	0.554 (0.438 - 0.671)	0.375	42.5	86.20%	32.30%
IL6_3	0.595 (0.484 - 0.707)	0.113	17.9	93.30%	30.00%
IL6_5	0.601 (0.492 - 0.711)	0.09	21	61.30%	61.00%
TNFα_0	0.635 (0.518 - 0.751)	0.031	14.8	64.30%	68.80%
TNFα_1	0.594 (0.473 - 0.714)	0.149	9.22	92.30%	29.50%
TNFα_3	0.57 (0.448 - 0.692)	0.264	22.3	44.40%	75.80%
TNFα_5	0.507 (0.38 - 0.635)	0.903	10.7	58.60%	55.60%
