# Supplementary material for: Effect of Perioperative Interleukin-6 and Tumor Necrosis Factor-α on Long-Term Outcomes in Locally Advanced Gastric Cancer: Results from the CLASS-01 Trial
Source: J Immunol Res. 2022 Jul 8;2022:7863480. doi: 10.1155/2022/7863480 (PMC9289757; doi:10.1155/2022/7863480)
Supplement: Supplementary 1 — Table 1: the analysis of ROC curve of the relationship between inflammatory markers and 5-year OS. Table 2: the association of IL6_5 levels with short-term outcomes. [file 7863480.f1.zip › 7863480.f1/Supplementary Table 2.pdf]

Supplementary Table 2 The association of IL6_5 levels with short-term outcomes

Factors	High IL6_5 (n, %)	Low IL6_5 (n, %)	P value
Total complications[a]	17 (29.3)	12 (16.4)	0.078
Pulmonary infections[b]	6 (10.3)	1 (1.4)	0.044
Lymphorrhagia[b]	3 (5.2)	4 (5.5)	1
Effusion or pyocelia[b]	4 (6.9)	2 (2.7)	0.405
Urinary tract infection[b]	1 (1.7)	2 (2.7)	1
Other[b]	4 (6.9)	4 (5.5)	0.732
Clavien–Dindo classification			
I[b]	1 (1.7)	2 (2.7)	1
II[a]	16 (23.6)	10 (13.7)	0.048
Hospital stay (days, median, IQR)[c]	11 (7.5-14)	9.5 (8-14.75)	0.982

a. Calculated by using χ^2 method.

b. Calculated by using Fisher’s exact method.

c. Calculated by using Mann-Whitney U.
