# Supplementary material for: Effect of Perioperative Interleukin-6 and Tumor Necrosis Factor-α on Long-Term Outcomes in Locally Advanced Gastric Cancer: Results from the CLASS-01 Trial
Source: J Immunol Res. 2022 Jul 8;2022:7863480. doi: 10.1155/2022/7863480 (PMC9289757; doi:10.1155/2022/7863480)

Supplementary Figure 1 The association of IL6_1, IL6_3, TNFα_1, TNFα_3 and TNFα_5 levels with DFS and OS in LAGC

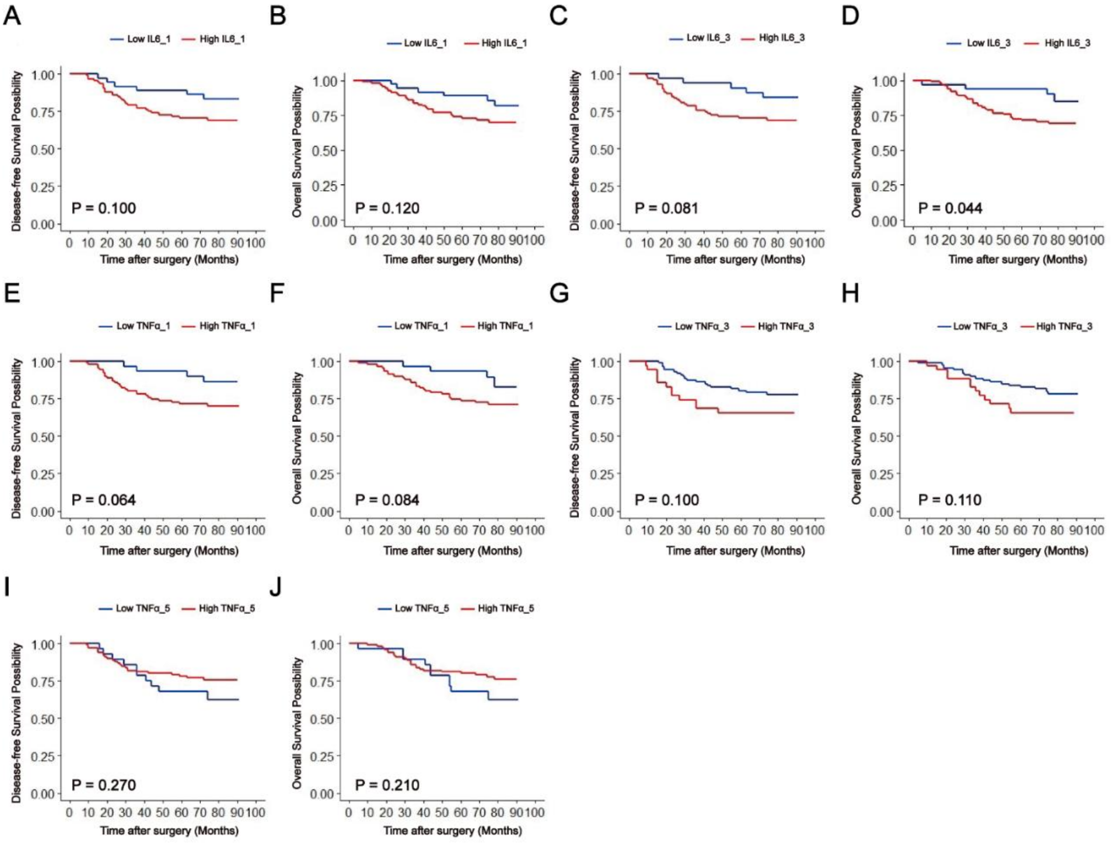

Supplement: Supplementary 2 — Figure 1: the association of IL6_1, IL6_3, TNFα_1, TNFα_3, and TNFα_5 levels with DFS and OS in LAGC. Figure 2: the nonlinear association between the serum IL6_0 and TNFα_0 levels with survival in LAGC patients. Figure 3: the stratified analysis of IL6_0, TNFα_0, and survival prognosis based on the TNM stage (stages I/II/III). [file 7863480.f2.zip › 7863480.f2/Supplementary Figure 1.pdf]
